# Partially inverted and degloved penile skin mimicking the appearance of female genitalia in cases of deaths caused by trains

**DOI:** 10.1007/s12024-024-00910-8

**Published:** 2024-12-02

**Authors:** Margaux Zarattin, Mohamed Yassine Braham, Jean-Loup Gassend

**Affiliations:** Centre Universitaire Romand de Médecine Légale, Centre Hospitalier Universitaire Vaudois, Lausanne, Switzerland

**Keywords:** Penis degloving, Train-related injuries, Forensic pathology

## Abstract

Death by collision with an incoming train is common in countries where a railroad network exists. In such cases, when there is severe pelvic trauma, the penis may be partially degloved and turned inside out. The inverted penile skin may then resemble a vulva and the scrotum may mimic labia majora, causing the injured male genitalia to strongly resemble female genitalia. Forensic pathologists should be aware of this possibility when they are called to such a scene of death and are asked by the police to immediately determine the sex of the victim. In the challenging circumstances of a chaotic on-site railway death investigation, an inexperienced doctor might easily mistake male genitals for female genitals and thus delay correct identification of the victim by the police.

## Images in forensics

**Fig. 1:**
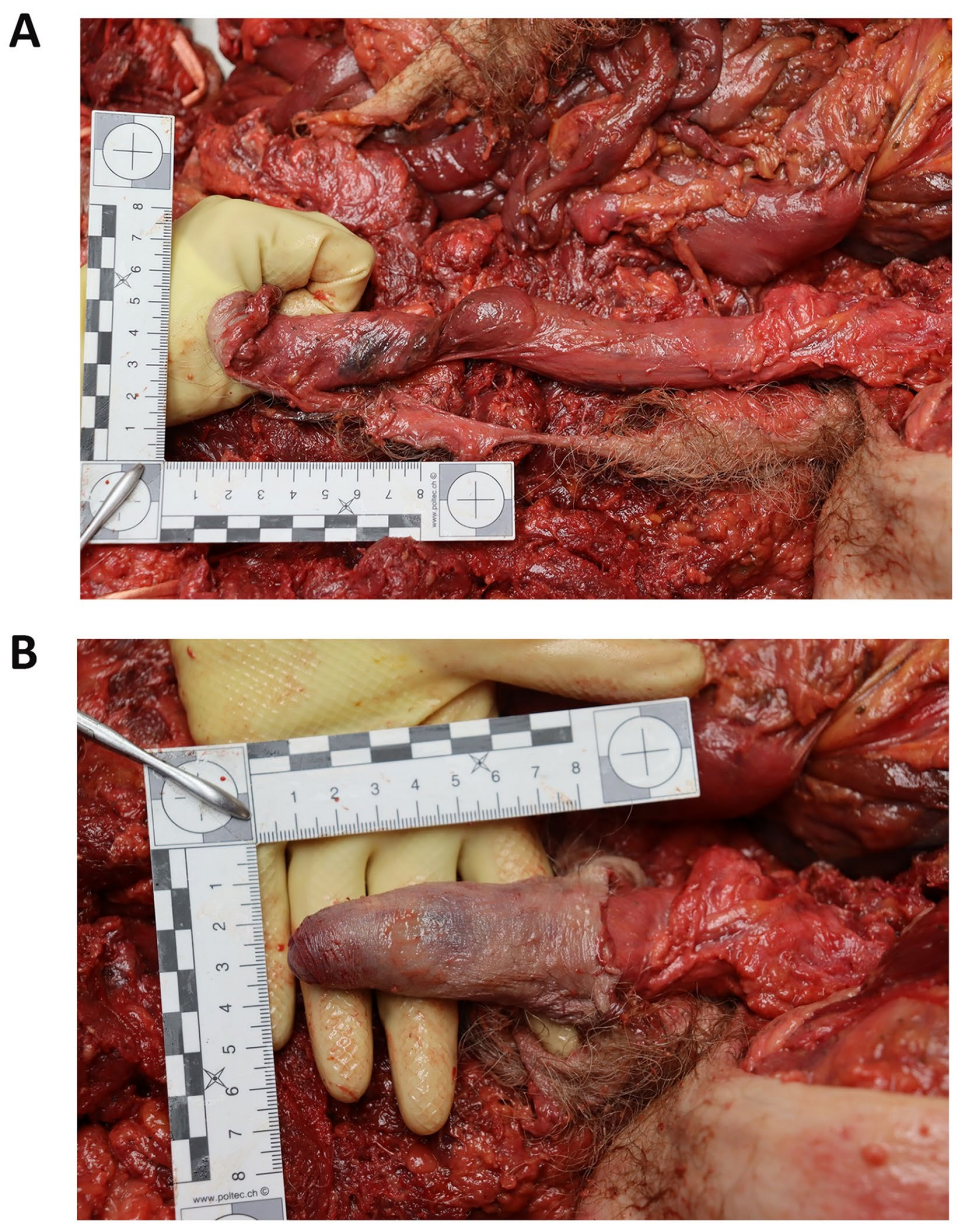
Typical example of a degloved penis as found, and with the skin put back into anatomical position. The doctor should carefullylook whether a structure resembling a degloved penis is present in the pelvic area, as seen in Fig. 1. It must be kept in mind that in deaths caused by a train, the degloved penis may be mangled or completely missing

**Fig. 2:**
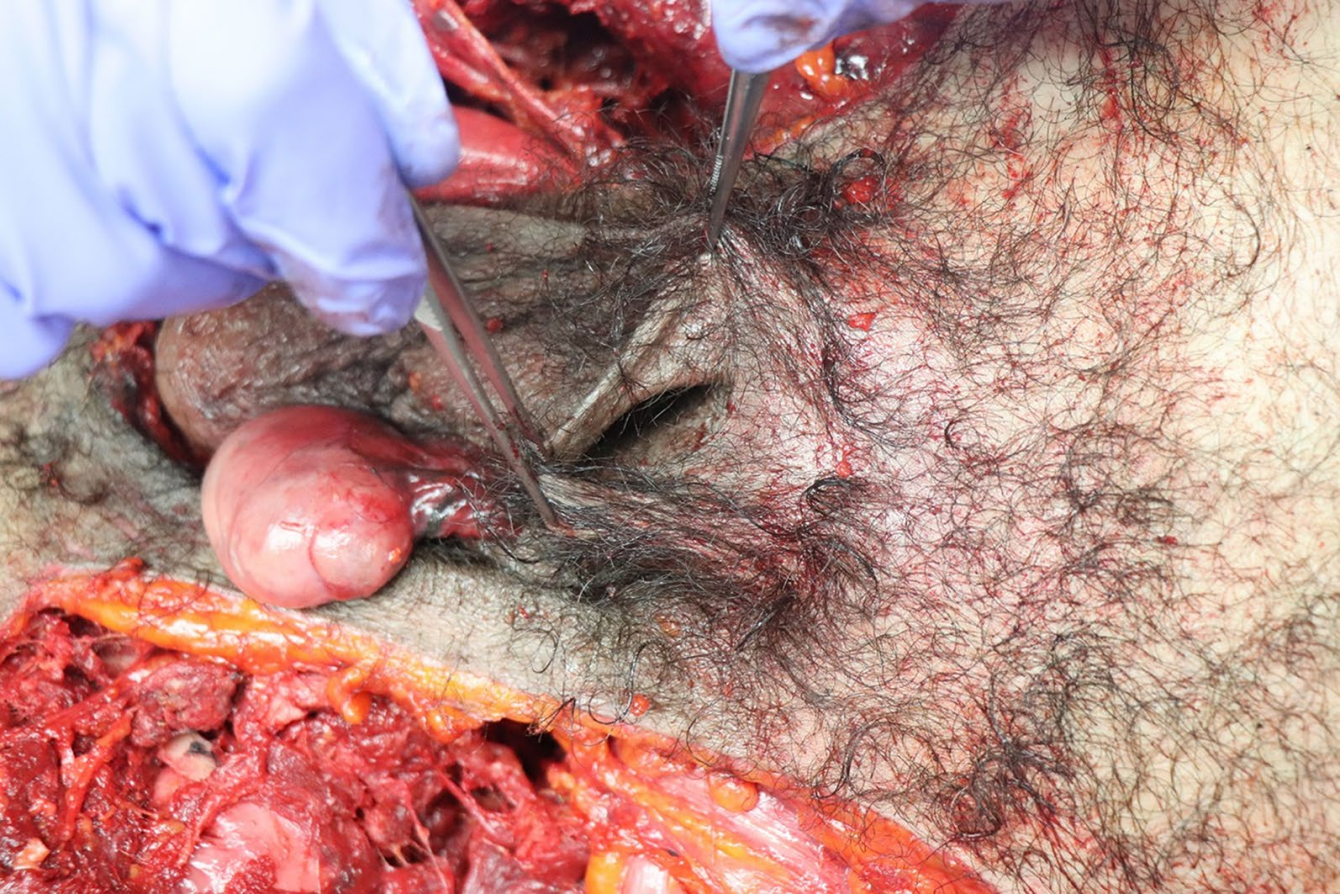
The “hole” that is formed when the penis is partially degloved and turned inside out

Worldwide, in locations in which a railroad network exists, a commonly used method of suicide is to jump, stand or lie in the path of an incoming train [1]. In Switzerland, over the past ten years, the number of suicides by train has varied between 101 and 149 per year. In 2022, of the 101 cases of suicide by train, more than half (66 cases) involved men [2]. Accidental deaths caused by trains are also common. Therefore, all the forensic pathologists at the University Center of Legal Medicine Lausanne-Geneva (Centre universitaire romand de médecine légale) frequently perform external examinations or autopsies on bodies of people who were killed by a collision with a train. The forensic pathologist on duty is also occasionally called to attend the scene of railway fatalities. In deaths caused by a moving train, the bodies are usually mangled, with injuries such as decapitation, amputations and evisceration of various organs being common [3]. In the preliminary phases of the police investigation, the identity of the victim may be unknown, and the severe injuries present on the body further complicate the identification procedure. The forensic pathologist on site may therefore be asked by the police to immediately give his opinion on the age and sex of the victim, and to search for any characteristic signs such as tattoos or surgical scars. Rapid determination of sex may consequently be crucial. The pelvic area is frequently severely injured in cases of train deaths [4]. It is not uncommon for the skin of the penis to be pulled aside along with a sub-amputated lower limb, while the root of the penis remains firmly attached to the pelvis. In such cases, the skin of the penis may be degloved from the base of the penis, while remaining attached to the distal penis, causing the penis skin to turn “inside out” like a sock (Fig. 1). After such an event, the degloved penis is no longer clearly visible on the backdrop of traumatized tissues, and the inverted penile skin leaves an apparent “hole” where the penis should be located (Fig. 2). By a similar mechanism, the testicles may be pulled out of the scrotum and the scrotum may be inverted.

**Fig. 3:**
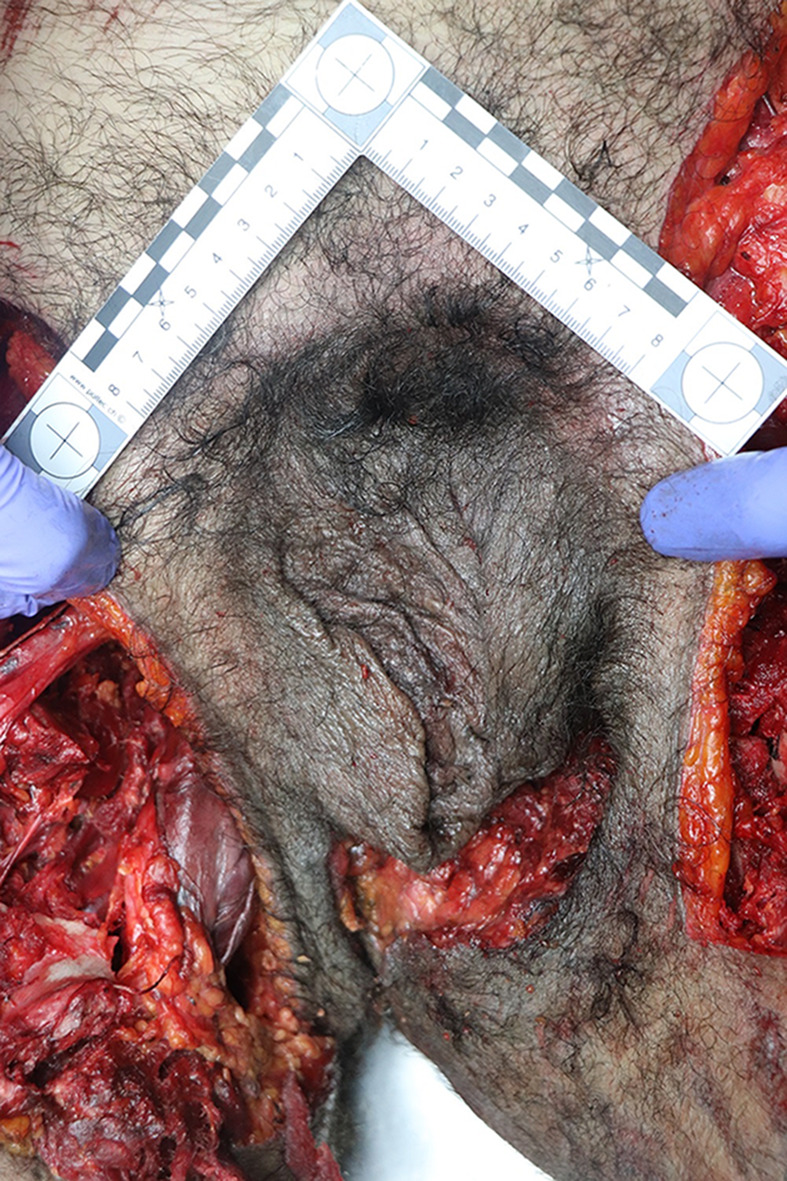
Degloved and inverted penis and partially inverted scrotum, making the male genitals strongly resemble female genitalia. The doctor should carefully examine the skin of the genital area in order to determine their true nature, such as pulling on the skin resembling a labia majora to determine if it may in fact be an inverted scrotum. In this case, the hairs have a distinct male distribution and density, however similar lesions on a male with fewer hairs could easily cause the body to be misidentified as female on cursory examination

Depending on the exact nature of the wounds, male genitalia may therefore not only be very difficult to recognize, they may furthermore strongly resemble female genitalia, the inverted penis skin resembling a vulva, and the scrotum mimicking the labia majora (Fig. 3). During the writing of this article, Fig. 3 was informally shown to several resident doctors of our center, all of which determined that they were looking at a photo of female genital organs.

It is notable that the surgical procedure used to achieve male to female sex change bears anatomical similarities with the traumatic mechanisms mentioned in the previous paragraphs, the penile skin being inverted by the surgeon in order to create the neo-vagina [5, 6]. In train-related deaths, forensic pathologists, emergency personnel and police should therefore be aware that very careful examination of the body may be required to determine sex in cases involving severe pelvic trauma. This difficulty is further compounded by the rather chaotic nature (bad weather, nighttime, urgency to reopen the tracks) of the on-scene investigations.

## Learning points

- Male genitalia may be degloved in a manner making them resemble female genitalia in cases of severe pelvic trauma associated with train deaths. Forensic pathologists called to the site of train deaths should therefore be careful before determining the sex of the victim if there is significant pelvic trauma.

## Data Availability

Not applicable.
